# Advanced intestinal regulation improves bowel preparation quality in patients with constipation: A systematic review and network meta-analysis

**DOI:** 10.3389/fphar.2022.964915

**Published:** 2023-01-24

**Authors:** Liang Ding, JinNan Duan, Tao Yang, ChaoQiong Jin, Jun Luo, Ahuo Ma

**Affiliations:** ^1^ Department of Gastroenterology, Shaoxing People’s Hospital, Shaoxing, Zhejiang, China; ^2^ Department of Infectious Diseases, Shaoxing People’s Hospital, Shaoxing, Zhejiang, China

**Keywords:** bowel preparation, colonoscopy, constipation, network meta-analysis, regimens

## Abstract

**Background:** Inadequate bowel preparation (IBP) has a critical influence on the colonoscopy procedure and is associated with significantly lower rates of detection of colorectal lesions. Constipation is an important risk factor of IBP, and some studies have attempted to address the bowel cleansing for constipated patients. However, there is still lack of consensus to guide the clinical work of bowel preparation (BP) for patients with constipation. Therefore, we aimed to perform a network meta-analysis to compare the overall efficacy of various regimens for BP in constipated patients.

**Methods:** We performed a comprehensive search of PubMed, MEDLINE, EMBASE, Cochrane, and Web of science to identify randomized controlled trials (RCTs) of bowel preparation regimens in constipated patients, update to January 2021. Two investigators independently evaluated articles and extracted data. The odds ratio (OR) with a 95% confidence interval (CI) was used to combine dichotomous data of the primary outcome which was defined as adequate bowel preparation (ABP). Rank probability was used to exhibit the outcome of the network meta-analysis.

**Results:** Eleven studies that included 1891 constipated patients were identified as suitable for inclusion. The proportion of ABP was associated with the administration of intensive regimen (OR 2.19, 95% CI 1.16–4.17, *p* = .02, I2 = 84%). Moreover, an intensive regimen had a significant efficacy and light heterogeneity when the same basic laxative program was used (OR 4.06, 95% CI 3.04–5.43, *p* < .0001, I2 = 0%). In the network meta-analysis, the protocol of a normal regimen + A (normal regimen plus advanced intestinal regulation) had a significant effect for bowel preparation compared with a normal regimen + IR (normal regimen plus irritating laxative regimen) (OR 5.21, 95% CI 1.18–24.55), H PEG (4L- polyethylene glycol) (OR 8.70, 95% CI 1.75–52.56), and normal regimen (NR) (OR 7.37, 95% CI 2.33–26.39). In the remaining protocols, no significant difference was observed in any comparison. No significant severe adverse events (AEs) associated with bowel preparation were reported in included studies.

**Conclusion:** Intensive regimens could improve bowel cleansing quality for patients with constipation, and advanced intestinal regulation regimens may be superior to others.

## 1 Introduction

Colonoscopy is considered the most valuable screening tool for gastrointestinal disease especially for colorectal cancer and precancerous lesions as successful colonoscopy can improve the mortality rate of colorectal cancer through detection and resection of tumors at an early and treatable stage; about every 1% increase in the adenoma detection rate will decrease 3% incidence and 5% mortality in colorectal cancer (Corley et al., 2014). The success of colonoscopy to find colorectal lesions is associated with the quality of the bowel visibility, and IBP significantly decreases the rate of detection of colorectal lesions with about .53 odds ratio in early adenomas and .74 odds ratio in advanced adenomas comparing inadequate with adequate bowel preparation (Sulz et al., 2016).

With a global prevalence of 15%, constipation is a manifestation gastrointestinal dysmotility in clinic, and its prevalence would steadily rise after the age of 50 years, which is the recommended age to perform colonoscopy for colorectal lesion screening (Bharucha and Lacy, 2020). However, constipation is an important risk factor for inadequate bowel preparation (IBP) and difficulty in colonoscopy which may lead to lesion missing, patient suffering, and time cost (Takahashi et al., 2005). A meta-analysis which included 67 studies and 75,818 patients finds that constipation adds the risk of IBP nearly up to twofold (Gandhi et al., 2018). There is little resolution when patients have IBP on the colonoscopy procedure, thus optimizing that the bowel preparation (BP) regimen is the critical measure to ensure the examination quality. In clinical practice, we empirically reinforce the BP program such as increasing laxative amount or adding adjuvants to address the BP of constipation, but the efficacy is under debate. Some RCT studies have been designed to verify the effect of “empirical” intensive regimens, and they provide some optional choices for clinical work (Hassan et al., 2019). However, these options have extremely diverse, and there is still lack of arbitrary and objective evidence to recommend a special regimen; even some RCTs have attempted to address the obstacle by comparing a series of BP regimens (Saltzman et al., 2015; Hassan et al., 2019).

Therefore, we aimed to perform a network meta-analysis as it allows us evaluating the indirectly comparative efficacy of multiple treatments in individual RCTs to determine the ideal bowel preparation regimen for constipated patients.

## 2 Methods

We performed a systematic review and network meta-analysis according to the Cochrane Handbook (https://training.cochrane.org/handbook) and reported according to Preferred Reporting Items (Liberati et al., 2009). The registration number is CRD42021238380 in PROSPERO. We claim that there is no ethical approval or patient consent was required.

Search methods

The databases of PubMed, MEDLINE, EMBASE, Cochrane, and Web of science were searched, update to January 2021. The search strategy identified in [All Fields] with the term: (prepar* OR clean*) AND (bowel* OR colon* OR intestin*) AND (colonoscopy) AND (constipat* OR fecal impaction), and the article type was restricted in “trail.”

### 2.1 Inclusion and exclusion criteria

Inclusion criteria: 1) studies were randomized controlled trials and report ABP, 2) subjects should be constipated adult patients (as diagnosed by a clinician, or using any recognized diagnostic criteria) that prepare to colonoscopy, 3) study purpose should be related with bowel preparation quality, 4) study interventions were pharmacological therapies, and 5) outcome should include dichotomous data about ABP.

Exclusion criteria: 1) studies not adhering to the inclusion criteria, 2) studies with only an abstract or commentary, and 3) studies that include other interventions like diet, education, and exercise.

### 2.2 Outcome assessment

The primary outcome is ABP, which is defined as follows: 1) total score more than 6 of the Boston Bowel Preparation Scale (BBPS), 2) total score less than 6 of the Ottawa Bowel Preparation Quality Scale (OBPS), 3) grade between 1 and 2 of the Aronchick Scale, and 4) grade 1 or 2 of the bowel preparation quality grading score.

The secondary outcome is the adverse events and tolerability of different bowel preparation regimens.

### 2.3 Data extraction

Two investigators independently extracted the intent-to-treat data from eligible studies. Disagreements were resolved by discussion with an additional reviewer. The data included the first author, country, publication years, recruitment criteria, intervention, assessment, sample size, age, sex, adequate preparation number, and adverse events.

### 2.4 Assessment of quality

Two independent investigators assess the methodological quality of the included studies, and disagreements will be resolved by consensus and discussion with a senior investigator. The Cochrane risk of bias tool will be used to assess the risk of bias at the individual study. Using this tool, studies will be classified to be at high, low, or unclear risk of bias based on seven items (https://training.cochrane.org/handbook): 1. random sequence generation, 2. allocation concealment, 3. blinding of participants, 4. blinding of outcome data, 5. incomplete data, 6. selective reporting, and 7. other biases.

### 2.5 Statistical analysis

The estimated effects of OR with 95% CI were used to evaluate dichotomous data by Review Manager version 5.3 (The Cochrane Collaboration, Oxford, UK). Heterogeneity was calculated with I2 statistics. A fixed-effects model was used only in I2< 50%. Single study deletion was used to assess the sensitivity of estimated effects.

Bayesian network meta-analysis with convergence estimate was used to compare all possible comparisons by Stata SE 15 (StataCorp. College Station, Texas, USA) and Gemtc (GitHub). The parameters of the network meta-analysis were set as follows: 4 of chains, 20,000 of the tuning iterations, 50,000 of simulation iterations, 10 of thinning interval, 10,000 of inference samples, and 2.5 of variance scaling factor. *p* < .05 was judged as statistically significant. If the study has two or more intervention arms, we divide the “shared” group into two or more equal groups (reasonably independent comparisons) according to the Cochrane handbook (https://training.cochrane.org/handbook).

## 3 Result

### 3.1 Search result

**FIGURE 1:**
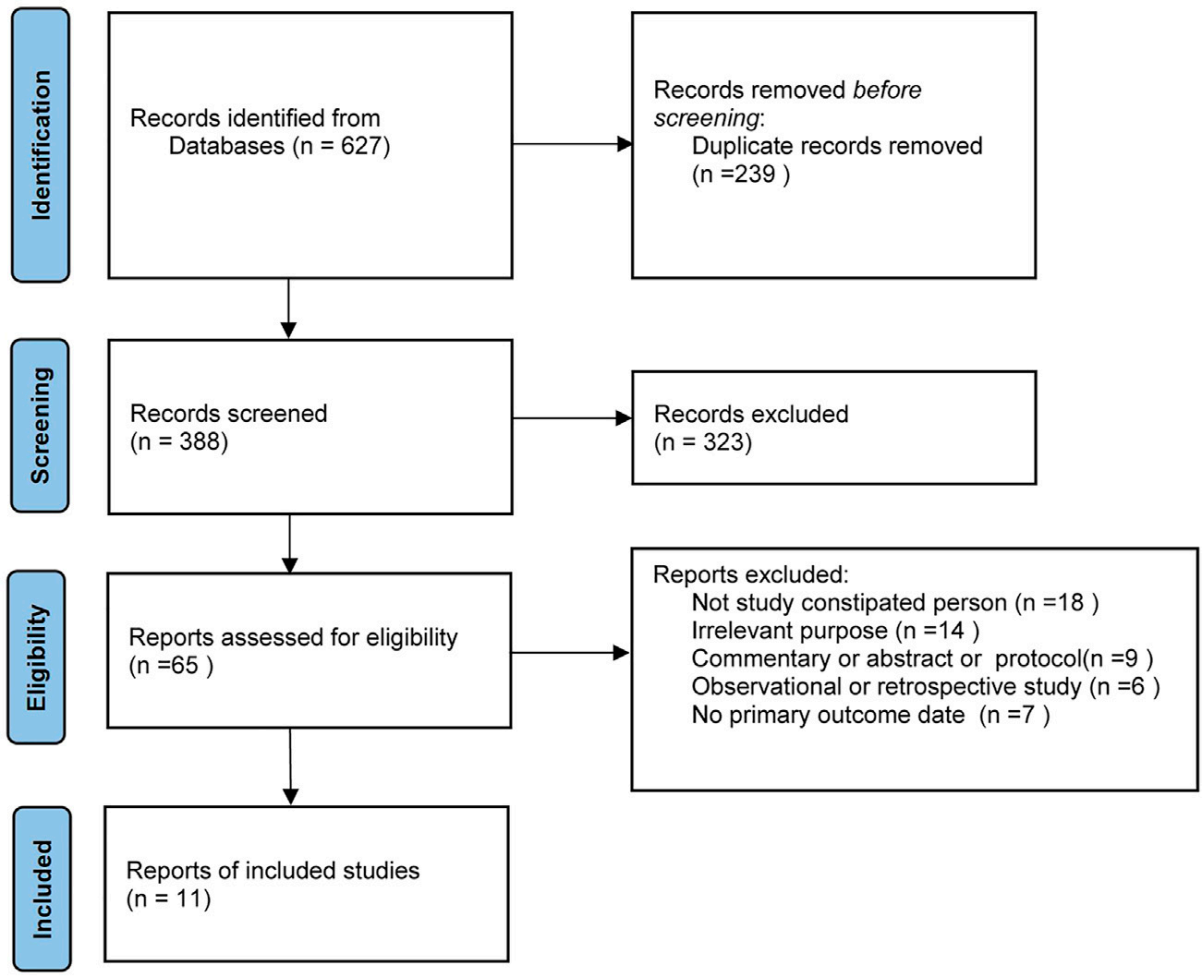
Prisma flowchart.

The search strategy identified a total of 627 citations; after removing 239 duplicates and 323 obviously irrelevant articles, we retrieved 65 articles for full-text appraisal (Figure 1). Finally, 11 articles were included in qualitative synthesis; 54 studies did not meet the inclusion criteria because of the reasons listed in Figure 1, most often because the participant was not a constipated person. It is worth mentioning that seven studies that included 868 patients were excluded because there was no primary outcome, and these study characteristics are shown in Supplementary Table S1.

### 3.2 Study characteristics

Fourteen regimens were studied for intestinal cleansing in 1,891 patients from 11 studies (Arezzo, 2000; De Salvo et al., 2006; Lee et al., 2010; Tajika et al., 2012; Tian et al., 2012; Pereyra et al., 2013; Parente et al., 2015; Li et al., 2017; Yu et al., 2018; Zhong et al., 2018; Chancharoen et al., 2019). The test groups usually choose the regimen of conventional laxatives plus additional stimulant laxatives, prokinetic drugs, or advanced intestinal regulation such as using probiotics and dietary fiber, and the control group usually chooses low-dose laxative such as 2 L polyethylene glycol (PEG) or sodium phosphate (NaP). More information about study characteristics is summarized in Table 1.

**TABLE 1:** Main characteristics of included studies.

Author	Year	Country	Constipation criterion	BP quality assessment	BP regimen	NP (f)	Age (M± SD)
Arezzo, A	2000	Germany	Asked if he or she experienced constipation	Non-validate score	Senna + MgSO_4_	40 (NA)	NA
					4L PEG	43 (NA)	NA
					NaP	48 (NA)	NA
De Salvo, L	2006	Italy	Rome II	Non-validate score	Senna + MgSO_4_	36 (NA)	61.4 ± 12.0
					2L PEG + bisacodyl	40 (NA)	60.5 ± 10.9
					NaP	25 (NA)	61.9 ± 12.6
Lee, H	2010	Korea	Rome III	BP quality grading	Probiotic 14 days + NaP	51 5)	40.5 ± 11.4
					NaP	53 5)	42.2 ± 11.7
Tajika, M	2012	Japan	<2 bowel movements per week more than a year	Aronchick’s criteria	Mosapride+2L PEG	16 (11)	67.3 ± 8.6
					2L PEG	5 4)	67.8 ± 10.1
Tian, Xia	2012	China	Asked if he or she experienced constipation	BP quality grading	Probiotic 3 days + mosapride 3 days +2L PEG	86 (40)	NA
					Mosapride 3 days +2L PEG	82 (44)	NA
					2L PEG	80 (42)	NA
Pereyra, Lisandro	2013	Argentina	Rome II	BP quality grading	NaP	22 (NA)	59 ± 13.2
					NaP + bisacodyl	20 (NA)	57 ± 11.1
					4L PEG	15 (NA)	60 ± 13.8
					2L PEG + bisacodyl	20 (NA)	59 ± 10.9
Parente, Fabrizio	2015	Italy	Rome III	OBPS	2L PEG + simethicone + citrates + bisacodyl	193 (105)	60 ± 13
					4L PEG	189 (113)	59 ± 14
Li, Y	2017	China	1 or 2 on the Bristol Stool Form Scale	BBPS	2L PEG + bisacodyl	234 (160)	52.1 ± 9.8
					2L PEG	233 (156)	52.2 ± 9.7
Yu, Z. B	2018	China	Rome III	BBPS	Lactulose 2 days+2L PEG	36 (NA)	NA
					2L PEG + senna	36 (NA)	NA
					2L PEG	36 (NA)	NA
Zhong, Shishun	2018	China	Chinese chronic constipation guide	BBPS	Testa triticum tricum 7 days +3L PEG	93 (61)	52.9 ± 12.3
					3L PEG	97 (60)	53.3 ± 12.6
Chancharoen, A	2019	Thailand	Rome III or a score of 1 or 2 on the Bristol Stool Form Scale	OBPS	4L PEG + pre-2L PEG	37 (23)	57.6 ± 9.4
					4L PEG	39 (25)	59.2 ± 7.2

BP, bowel preparation; BBPS, Boston Bowel Preparation Scale; OBPS, Ottawa Bowel Preparation Scale; NaP, sodium phosphate; PEG, polyethylene glycol; NA, not available; NP, number of patients; f, female; M±SD, mean ± standard deviation.

### 3.3 Risk of bias in included studies

**FIGURE 2:**
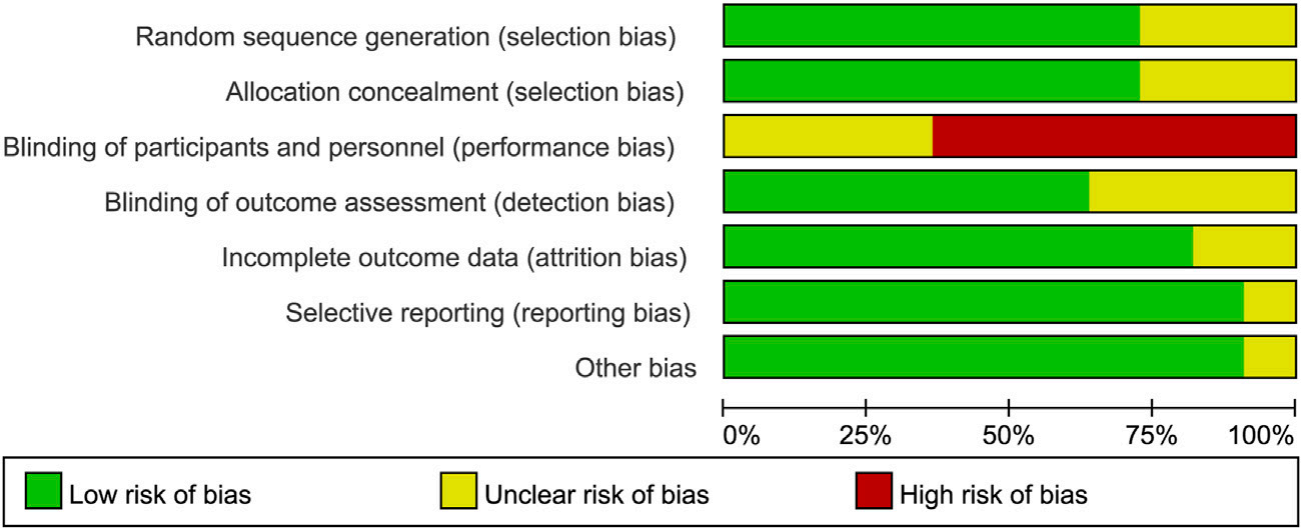
Risk of bias graph: review authors’ judgements about each risk of bias item presented as percentages across all included studies.

A majority of bias items showed low risk and unclear risk, but most studies showed a high risk in the item of performance bias since the fact that experimenters or medical workers must give the details of a BP plan to ensure the compliance of participants. More details are shown in Figure 2 and Supplementary Table S2.

### 3.4 Direct meta-analysis

**FIGURE 3:**
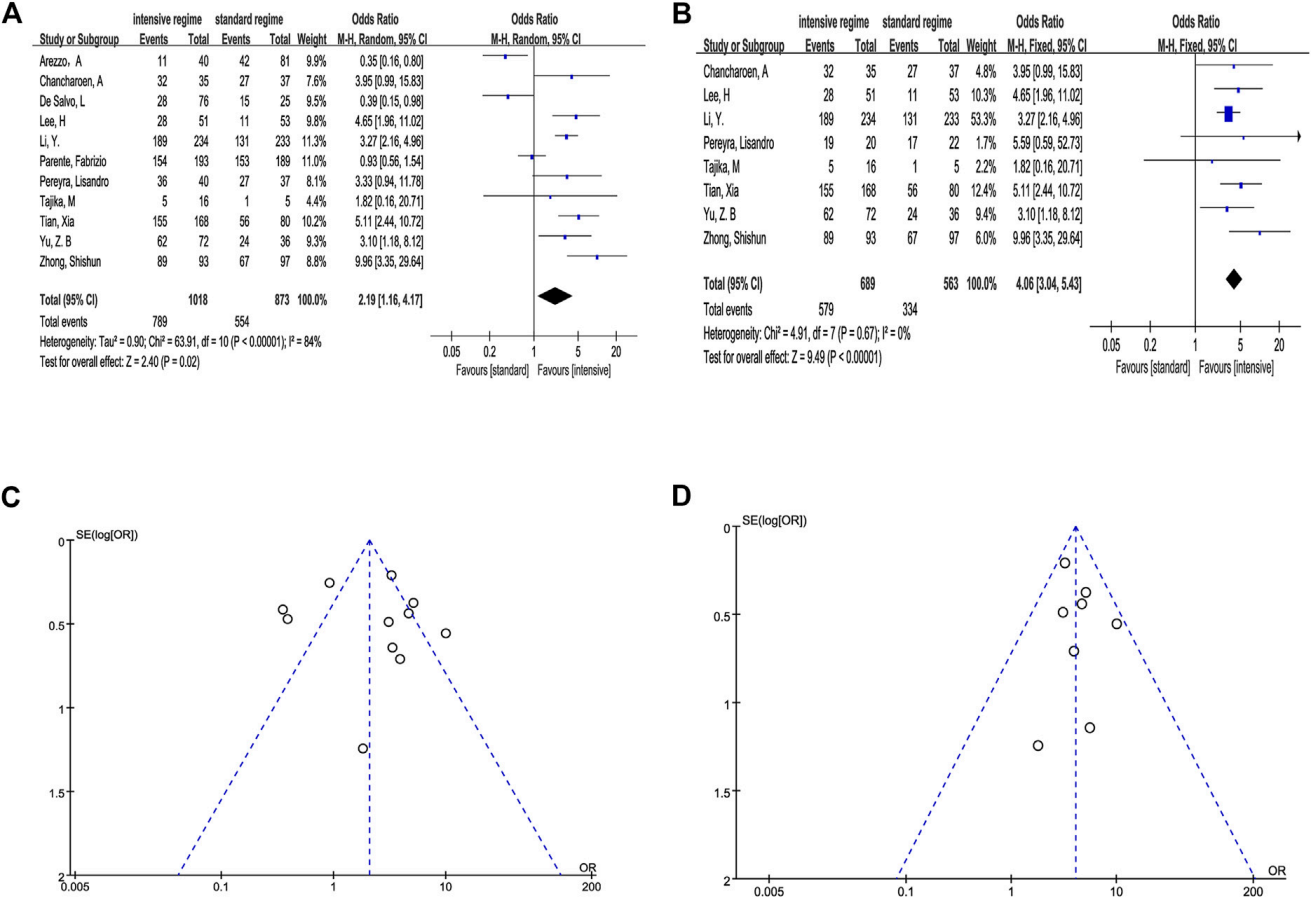
Outcome of direct meta-analysis. **(A, C)** Forest graph and funnel graph of intensive regimen vs. standard regimen. **(B, D)** Forest graph and funnel graph of intensive regimen vs. standard regimen based on the same laxative.

We first divided the BP regimens into “intensive regimen” (combination of extra preparation program with conventional single laxative) and “standard regimen” (conventional single laxative like PEG and NaP). In total, 789 (77.5%) participants in the intensive group achieved ABP and 554 (63.5%) in the standard group. The estimated effect for the primary outcome was significantly higher in the intensive regimen (OR 2.19, 95% CI 1.16–4.17, *p* = .02, I2 = 84%) (Figure 3A).

We observed that the studies of Arezzo (2000), De Salvo et al. (2006), and Parente et al. (2015) conducted a completely different program in comparison which is not consistent in the use of laxative, and Pereyra et al. (2013) also had the same question in a certain way. Hence, we performed the analysis based on the same basic laxative program (4L PEG, 2L PEG or NaP) after removing inconsistent data. The result still indicated that the intensive regimen has a significant efficacy compared with the standard regimen when the same basic laxative program was used (OR 4.06, 95% CI 3.04–5.43, *p* < .0001, I2 = 0%) (Figure 3B).

Sensitivity analysis proved that all estimate effect maintained stability in the process of single study deletion (Supplementary Table S3).

We seemingly observed potential asymmetry in the funnel plots (Figures 3C,D). In order to further evaluate the publication bias, we conducted Begg’s and Egger’s tests, and the results suggested no evidence proving publication bias (*p* > .1) (Supplementary Table S4).

### 3.5 Network meta-analysis

#### 3.5.1 Regimens and sample size

In order to further explore the efficacy of different schemes, we classified them into seven types according to the mechanisms and eliminated the program of Senna + MgSO_4_ because its effectiveness was as low as 27.6%. The seven types include normal regimen such as using low-volume PEG or NaP (NR), NR plus irritating laxative regimens (NR + IR), NR plus advanced intestinal regulation (NR + A), PEG plus prokinetic agents (PEG + P), PEG plus advanced intestinal regulation and prokinetic agents (PEG + A + P), high-volume PEG (H PEG), and H PEG plus once pre-using of PEG (H+ pre-PEG).

**FIGURE 4:**
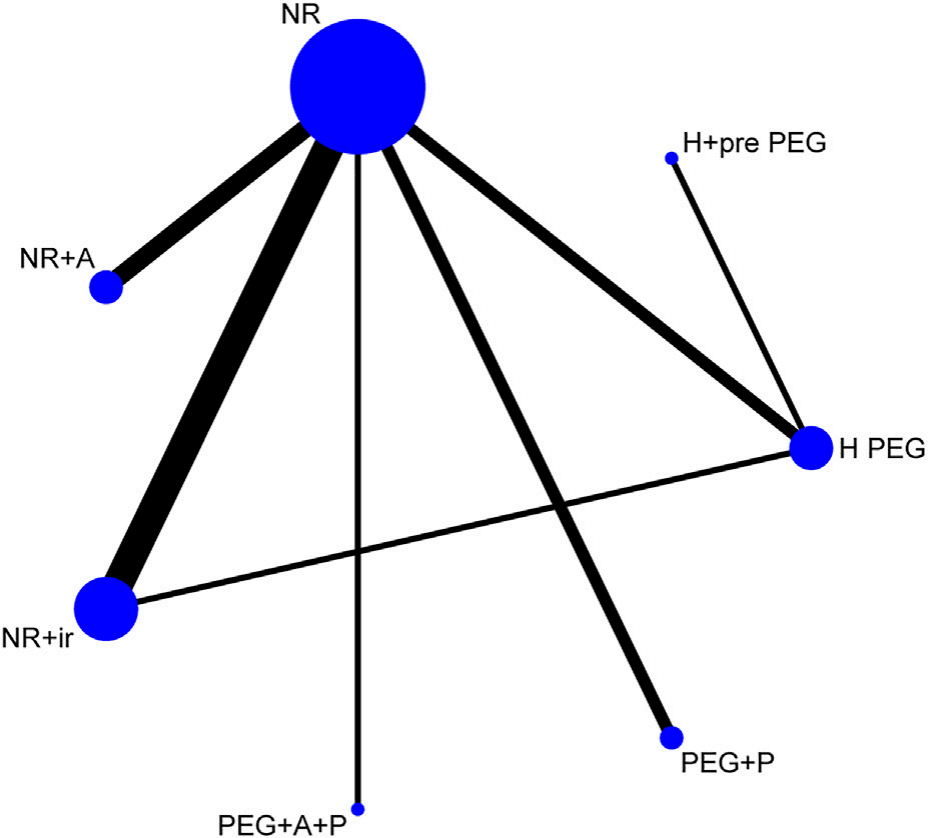
Network of comparisons for the Bayesian meta-analysis. Each circle represents a type of regimen. Each line represents direct comparison between two regimens. The width of the linking line is proportional to the number of studies. NR, normal regimen such as using low-volume PEG or NaP; NR + IR, NR plus irritating laxative regimens; NR + A, NR plus advanced intestinal regulation; PEG + P, PEG plus prokinetic agents; PEG + A + P, PEG plus advanced intestinal regulation and prokinetic agents; H PEG, high-volume PEG; H + pre-PEG, H PEG plus once pre-using of PEG.

The sample size and comparisons of each regimen showed the network map that was made by Stata software (Figure 4). The circle represented different regimens, and the size of the circle was proportional to the regimen sample. The lines indicated direct comparisons between regimens, and the thickness of the line was proportional to the weight of each regimen comparing others.

#### 3.5.2 Quality assessment

The result of inconsistency factors was .53 with 95% CI .84–2.40, and the Random Effects Standard Deviation of Consistency model and the Inconsistency model kept a good consistency (.70 with 95% CI 0.31–1.51, .65 with 95% CI 0.22–1.47, respectively). Hence, we selected a consistency model for the network meta-analysis. In addition, a favorable convergence efficiency was provided by all PSRF values which were limited to 1.

### 3.6 Efficacy and rank probability analysis

Except for NR + A, any comparison of remaining six regimens showed no significant difference in the ABP rate. However, we found that the NR + A regimen showed significant superior efficacy than NR + IR (OR 5.21, 95% CI 1.18–24.55), H PEG (OR 8.70, 95% CI 1.75–52.56), and NR (OR 7.37, 95% CI 2.33–26.39) (Table 2).

**TABLE 2:** Network meta-analysis of adequate bowel preparation.

NR + A	PEG + A + P	H + pre-PEG	PEG + P	NR + IR	H PEG	NR
1.01 (.10, 11.29)						
1.97 (.12, 32.99)	1.94 (.07, 48.21)					
2.16 (.28, 17.20)	2.09 (.17, 28.98)	1.10 (.05, 21.70)				
5.21 (1.18, 24.55)	5.14 (.56, 48.51)	2.66 (.21, 48.70)	2.43 (.38, 16.92)			
8.70 (1.75, 52.56)	8.66 (.87, 96.40)	4.42 (.49, 45.30)	4.09 (.55, 33.15)	1.67 (.49, 6.02)		
7.37 (2.33, 26.39)	7.43 (.96, 55.09)	3.80 (.32, 48.70)	3.47 (.68, 19.02)	1.43 (.59, 3.41)	.85 (.24, 2.71)	

NR, normal regimen such as using low-volume PEG or NaP; NR + IR, NR plus irritating laxative regimens; NR + A, NR plus advanced intestinal regulation; PEG + P, PEG plus prokinetic agents; PEG + A + P, PEG plus advanced intestinal regulation and prokinetic agents; H PEG, high-volume PEG; H + pre-PEG, H PEG plus once pre-using of PEG.

Effect estimates presented as odds ratio (OR) with 95% confidence interval (CI).

*p*value < .05 are in bold.

In the ranking table, NR + A (33% with Rank 1 and 39% with Rank 2) and PEG + A + P (38% with Rank 1 and 27% with Rank 2) had priority of ranking top; other probability data are shown in Table 3.

**TABLE 3:** Rank probability of bowel preparation regimens.

Regimen	Rank 1	Rank 2	Rank 3	Rank 4	Rank 5	Rank 6	Rank 7
NR + A	.33	.39	.2	.07	.01	0	0
PEG + A + P	.38	.27	.2	.1	.02	.01	.02
H + pre-PEG	.2	.16	.23	.21	.07	.07	.06
PEG + P	.09	.17	0.3	.29	.08	.04	.03
NR + IR	0	.01	.05	.23	.46	.18	.06
H PEG	0	0	.01	.05	.15	.28	.5
NR	0	0	.01	.05	.21	.41	.32

NR, normal regimen such as using low-volume PEG or NaP; NR + IR, NR plus irritating laxative regimens; NR + A, NR plus advanced intestinal regulation; PEG + P, PEG plus prokinetic agents; PEG + A + P, PEG plus advanced intestinal regulation and prokinetic agents; H PEG, high-volume PEG; H + pre-PEG, H PEG plus once pre-using of PEG.

### 3.7 Adverse events and tolerability with bowel preparation

In general, each regimen has a low rate of AEs and good tolerability although the definition was not according to a homogeneous standardized role. Only one serious adverse event (intestinal occlusion) was reported in the 4L PEG group, but it was not considered as the cause of colonic lavage solution (Parente et al., 2015). Other AEs mainly include gastrointestinal symptoms and are often mild and transient. Statistically significant difference was not obvious for secondary outcomes, except vomiting (OR 0.60, 95% CI 0.38–.97) (Table 4). However, we found that the AEs of vomiting were rare, and sensitivity analysis indicated two studies (Tajika et al., 2012; Zhong et al., 2018) which contributed to the major advantage of vomiting, meaning the difference was unstable.

**TABLE 4:** Secondary outcomes of intensive regimen vs. standard regimen.

Outcome	N1 (E/S)	N2 (E/S)	OR	95% CI	I2 (%)
Nausea	115/902	135/767	.75	.57–1.00	19
Vomiting	32/709	45/578	.60	.38–.97	11
Bloating	96/902	101/767	.72	.40–1.30	61
Abdominal pain	70/902	50/767	1.21	.83–1.79	32
Tolerability	786/874	600/723	1.19	.56–2.54	68

N1, number of intensive regimens; N2, number of standard regimens; E/S, event/sample size; OR, odds ratio; CI, confidence interval; I2 >50% indicates high heterogeneity.

## 4 Discussion

Constipation is a frequent risk factor of poor BP quality, and empirically strengthening bowel cleansing is a common clinical coping strategy (Hassan et al., 2019). In the article, we systematically investigated the efficacy and safety of the additional BP program for constipated patients to find the best solution. To our knowledge, this study is the first conducted network meta-analysis to address this clinical problem.

Two reviewers independently undertook a contemporaneous and exhaustive literature search which included searching the “grey” literature and clinicaltrials.gov, and recruited 11 studies which provide binary information about ABP or IBP but excluded seven articles that only provide the bowel score. The main reason for our choice is that IBP is more clinically meaningful premonition in the omission of intestinal lesions than the difference of bowel score (Clark et al., 2014). In addition, there was substantial variation in the definition of the bowel cleansing score which will cause great heterogeneity in the statistical process.

In total, 1,891 patients were studied for intestinal cleansing from 11 studies. To our knowledge, this is the largest meta-analysis of bowel preparation in patients with constipation. In the traditional meta-analysis, our results found that intensive regimens could acquire a high rate of ABP (OR 2.19, 95% CI 1.16–4.17) although there is major heterogeneity (I2 = 84%). Moreover, pairwise results also exhibited significant superiority (OR 4.06, 95% CI 3.04–5.43) in intensive regimens but only with light heterogeneity (I2 = 0%) when we eliminate the studies that compare double intervention factors: intensive measure and inconsistent laxative, meaning that large heterogeneity comes from the difference of basic purgative. As a control group, the “standard regimen” includes the two most common laxatives in clinical practice (Parra-Blanco et al., 2014; Hassan et al., 2019): PEG scheme and NaP scheme that indicate that constipated patients could usually benefit from extra bowel preparation in practice. Meanwhile, all intensive regimens show the same safety as the standard regimen despite the fact that adverse events were not reported according to a homogeneous standardized role. Those outcomes are inspiring and therefore likely to be important in medical field, in order to help inform treatment decisions.

However, it remains confusing until we explain the effectiveness of the specific regimen. Thus, in the subsequent Bayesian network meta-analysis, which integrates the superiority of direct and indirect evidence, we tried to explore the effects of different regimens for prokinetics, intestinal regulation, combined stimulant laxatives, and high-dose laxative regimens. The first enlightening result is that NR + A, PEG + A + P, and H+ pre-PEG had the three top ranks which indicated that advanced intestinal regulation or pre-bowel preparation could maximize the benefits of cleansing quality for constipation. Five RCTs provide five different pre-bowel preparations including PEG (Chancharoen et al., 2019), lactulose (Yu et al., 2018), testa triticum (Zhong et al., 2018), and two probiotic products (*Bacillus subtilis* and *Streptococcus faecium* in the study of Lee et al. (2010) and *Clostridium butyricum* in the study of Tian et al. (2012)). Both lactulose and PEG which could increase the water amount of stool are commonly used osmotic laxatives in the treatment of chronic constipation (Lee-Robichaud et al., 2010). By taking additional laxatives on the basis of the standard bowel preparation program, RCTs of both PEG (Chancharoen et al., 2019) and lactulose (Yu et al., 2018) exhibit improvement in the bowel quality in constipation. In addition, a multicenter retrospective study from Japan found that the improved rate by using short-duration PEG was 72.6% for chronic constipation whose previous bowel preparation had been fair or poor (Yoshida et al., 2020). Despite differences in results, at least on some options, dietary fiber and probiotics were commonly considered as functional supplements/food that could improve defecation in constipated patients (Ford et al., 2014). Correspondingly, the three RCTs (Lee et al., 2010; Tian et al., 2012; Zhong et al., 2018) in our study demonstrate that taking probiotics or dietary fiber in advance can improve the quality of bowel preparation. However, in view of the huge and complex of human flora, the role of probiotics, prebiotics, and synbiotics in intestinal function is still being further explored. For example, insoluble fibers can increase a regulatory stool frequency but wheat dextrin and finely ground wheat bran would decrease stool water content and bowel sensation, potentially aggravating constipation symptoms (Gill et al., 2021). Therefore, raising knowledge of functional supplements/food with different characteristics may help us choose the ideal product for prebowel preparation.

Another issue worth exploring is the duration of constipation management since preparation time ranges from 1 day to 2 weeks. It seems to be mechanism oriented, because high-dose laxatives can empty the intestines in a short time, while low-dose laxatives or functional supplements need more time to adjust intestinal function. Considering patient tolerance, symptom-oriented management may be able to guide the preparation time because if patients feel that difficult defecation is relieved, they are more likely to achieve qualified bowel preparation (Safder et al., 2008). Unfortunately, there is a lack of enough explanation of specific relationship between the disease severity and bowel preparation. The RCTs in our study also provide insufficient information about baseline characteristics and improvement degree of constipation. A few studies prove that bowel symptoms such as type 1 or 2 of the Bristol stool form scale (BSFS), starting-to-defecation interval ≥4 h, and infrequent bowel movement (<3/week) could predict IBP (Lee et al., 2017; Guo et al., 2020). Some studies found that the colon transit time test represents a useful mean for predicting IBP before colonoscopy (Park et al., 2015; Zhai et al., 2019). These evidences may be valuable for formulating individualized BP strategies in constipated patients until verified by a large cohort study. Regardless of the gap, our network meta-analysis intends to that reasonable symptom management before colonoscopy could maximize bowel cleansing to reach the standard of “adequate” for patients with constipation.

Abundant evidence indicates that high-volume PEG could provide the highest quality preparation (Johnson et al., 2014). However, volume-related discomfort and unpleasant taste may hinder the acceptability. Considering the limitations, several studies have suggested that low-volume PEG plus adjuvants such as ascorbate, citrate, and sports drinks may have the potentiality of addressing the issues under certain conditions (Saltzman et al., 2015; Hassan et al., 2019). Parente et al. (2015) discussed the role of previous two measures in patients with constipation, and they found that 2L PEG plus adjuvants perform equivalent in terms of bowel cleansing but better in patient tolerability and compliance. In our study, we observed that increasing the amount of PEG may be ineffective in constipation according to the data in table2. We speculate that high-dose laxatives may not be able to fully empty the constipated intestines in a short time because of colonic sensorimotor disturbances and pelvic floor dysfunction. However, since there is no direct comparison, it remains unclear if this is because of insufficient data, mix factor, or equivalent outcomes. In summary, we need head-to-head experiments and related mechanism examine to confirm the hypothesis.

One of the strengths in our study is that we help address clinical needs in practical settings by traditional and network meta-analysis. Another strength is the strict quality control of statistics which include mild heterogeneity, insignificant publication bias, and good index of sensitivity, consistency, and convergence, meaning that all results have a good credibility. There also are several limitations in the present study. First, there are differences in the preparation process, severity of symptom, and the endpoint used to define ABP. These are inevitable weakness in any meta-analysis because of the difference in individual trials which means we need to cautiously interpret outcomes even in mild heterogeneity (Nakagawa et al., 2017). Second, some studies may be underpowered owing to the relatively small sample. Third, inadequate participants blinding may elicit bias and impact the accuracy of the estimate. Fortunately, single-blind trials are more likely to influence the outcome of subjectively reported. In the definition of bowel cleanliness, it is more important to keep the endoscopists blind before observation. Our network meta-analysis may be criticized due to the absence of direct comparisons between most arms that may lead to confounding due to underlying differences, but the universality of various preparations raises the results of consistency and convergence that increase the credibility of outcome (Cipriani et al., 2013). In addition, our results tend to provide principled guidance for clinical decision-making, but the specific selection still needs to rely on the individualized characteristics of the patient.

In summary, we found that the intensive regimen and advanced intestinal regulation could increase the ABP rate, but increasing the amount of PEG may be ineffective in patients with constipation. Further checking the relationship between constipation severity/improvement and bowel preparation quality will help policy-makers refine clinical guidelines so that health-care providers can more efficiently and effectively develop a bowel cleaning strategy for constipated patients.

## Data Availability

The original contributions presented in the study are included in the article/Supplementary Material, further inquiries can be directed to the corresponding author.
