# Supplementary figures and images for: Advanced intestinal regulation improves bowel preparation quality in patients with constipation: A systematic review and network meta-analysis
Source: Front Pharmacol. 2023 Jan 24;13:964915. doi: 10.3389/fphar.2022.964915 (PMC9904507; doi:10.3389/fphar.2022.964915)

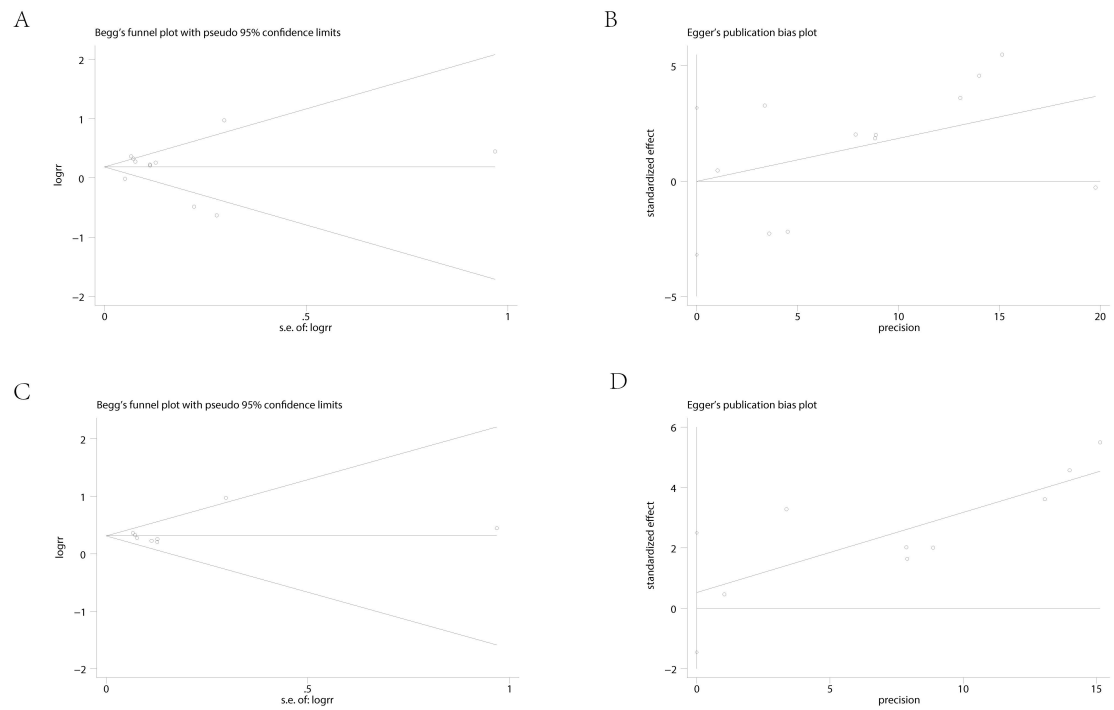

Supplement 4: figure of Begg's and Egger's test

Supplement: Supplementary file 1 [file DataSheet2.pdf]
