# Supplementary material for: Advanced intestinal regulation improves bowel preparation quality in patients with constipation: A systematic review and network meta-analysis
Source: Front Pharmacol. 2023 Jan 24;13:964915. doi: 10.3389/fphar.2022.964915 (PMC9904507; doi:10.3389/fphar.2022.964915)
Supplement: Supplementary file 2 [file Table1.docx]

| **Supplement 1:** Summary of no primary outcome studies. . | | | | | | | | |
| --- | --- | --- | --- | --- | --- | --- | --- | --- |
| **Author** | **Year** | **country** | **Constipation diagnosis criterion** | **BP assessment** | **BP regimen** | **Number (% female)** | **age (M±SD)** | **Outcome** |
| Chen, H | 2009 | china | Rome III | BP quality grading | 90ml NaP | 47 (51.1%) | 55.7±13.7 | 90ml NaP＞3L PEG |
|  |  |  |  |  | 3L PEG | 51 (52.9%) | 56.2±15.0 |  |
| Ren, L | 2013 | china | Chinese chronic constipation guide | BBPS | pre±1d Itopride +2L PEG | 38 | NA | pre±1d Itopride +2L PEG＞Itopride +2L PEG 2L PEGno significance in Itopride +2L PEG and 2L PEG |
|  |  |  |  |  | Itopride +2L PEG | 37 | NA |  |
|  |  |  |  |  | 2L PEG | 38 | NA |  |
| Zeng, J | 2013 | china | Chinese chronic constipation guide | BBPS | pre±1d Mosapride+2LPEG | 30 (66.7%) | 55.8±11.0 | pre±1d Mosapride+2LPEG＞Mosapride+2LPEG＞2L PEG |
|  |  |  |  |  | Mosapride+2LPEG | 30 (60%) | 56.4±10.9 |  |
|  |  |  |  |  | 2LPEG | 30 (66.7%) | 54.3±9.8 |  |
| Liu, Xiao | 2016 | china | constipation history | BBPS | 3d Procapride +2LPEG | 29 | NA | 3d Procapride +2LPEG and Procapride +2LPEG＞2L PEG no significance in 3d Procapride +2LPEG and Procapride |
|  |  |  |  |  | Procapride +2LPEG | 11 | NA |  |
|  |  |  |  |  | 2LPEG | 33 | NA |  |
| Lu, J | 2016 | china | Rome III | OBPS | 30mL lactulose solution +2L PEG | 45 | 46±3.6 | 30mL lactulose solution +2L PEG＞2L PEG |
|  |  |  |  |  | 2LPEG | 45 | 46±3.6 |  |
| Yildar, M | 2017 | Turkey | Rome III | BBPS | Sennoside calcium salt+pre±1h 210mL enema | 78 (53.8%) | 55.1±12.5 | only improve right colon Boston Bowel Preparation Scale score in pre±using enema |
|  |  |  |  |  | Sennoside calcium salt+210mL enema | 78 (56.4%) | 55.6±11.9 |  |
|  |  |  |  |  | Sennoside calcium salt | 71 (54.9%) | 55.6±11.1 |  |
| Xi, X | 2019 | china | Rome IV | BBPS | 60ml olive oil+3LPEG | 60 (65.0%) | 46.25±13.25 | using olive oil only improve right colon Boston Bowel Preparation Scale score |
|  |  |  |  |  | 1.5LPEG+60ml olive oil+1.5LPEG | 58 (56.9%) | 49.25±12.71 |  |
|  |  |  |  |  | 3L PEG | 59 (61.0%) | 45.76±12.79 |  |

BP: Bowel Preparation, BBPS: Boston Bowel Preparation Scale, OBPS: Ottawa Bowel Preparation Scale, NaP: sodium phosphate, PEG: polyethylene glycol, NA: not available
