# Supplementary material for: Advanced intestinal regulation improves bowel preparation quality in patients with constipation: A systematic review and network meta-analysis
Source: Front Pharmacol. 2023 Jan 24;13:964915. doi: 10.3389/fphar.2022.964915 (PMC9904507; doi:10.3389/fphar.2022.964915)
Supplement: Supplementary file 3 [file Table2.pdf]

	Random sequence generation (selection bias)	Allocation concealment (selection bias)	Blinding of participants and personnel (performance bias)	Blinding of outcome assessment (detection bias)	Incomplete outcome data (attrition bias)	Selective reporting (reporting bias)	Other bias
Arezzo，A	?	?	−	+	+	+	+
Chancharoen, A	+	+	?	+	+	+	+
De Salvo, L	?	?	−	?	+	+	+
Lee, H	+	+	?	+	+	+	+
Li, Y.	+	+	−	+	+	+	+
Parente, Fabrizio	+	+	−	+	+	+	+
Pereyra, Lisandro	+	+	?	+	+	+	+
Tajika, M	+	+	?	+	?	+	+
Tian, Xia	?	?	−	?	+	+	?
Yu, Z. B	+	+	−	?	?	?	+
Zhong, Shishun	+	+	−	?	+	+	+

Supplement2：Risk of bias in included studies
