# Supplementary material for: Advanced intestinal regulation improves bowel preparation quality in patients with constipation: A systematic review and network meta-analysis
Source: Front Pharmacol. 2023 Jan 24;13:964915. doi: 10.3389/fphar.2022.964915 (PMC9904507; doi:10.3389/fphar.2022.964915)
Supplement: Supplementary file 4 [file DataSheet1.pdf]

supplement 3: Sensitivity analysis prove that all estimate effect maintained the stable in the process of single study deletion .

Study or Subgroup	intensive regime Events	intensive regime Total	standard regime Events	standard regime Total	Weight	Odds Ratio M-H, Fixed, 95% CI
Chancharoen, A	32	35	27	37	0.0%	3.95 [0.99, 15.83]
Lee, H	28	51	11	53	10.8%	4.65 [1.96, 11.02]
Li, Y.	189	234	131	233	56.0%	3.27 [2.16, 4.96]
Pereyra, Lisandro	19	20	17	22	1.8%	5.59 [0.59, 52.73]
Tajika, M	5	16	1	5	2.3%	1.82 [0.16, 20.71]
Tian, Xia	155	168	56	80	13.0%	5.11 [2.44, 10.72]
Yu, Z. B	62	72	24	36	9.9%	3.10 [1.18, 8.12]
Zhong, Shishun	89	93	67	97	6.3%	9.96 [3.35, 29.64]
**Total (95% CI)**		**654**		**526**	**100.0%**	**4.07 [3.03, 5.47]**
Total events	547		307			

Heterogeneity: Chi² = 4.91, df = 6 (P = 0.56); I² = 0%
Test for overall effect: Z = 9.30 (P < 0.00001)

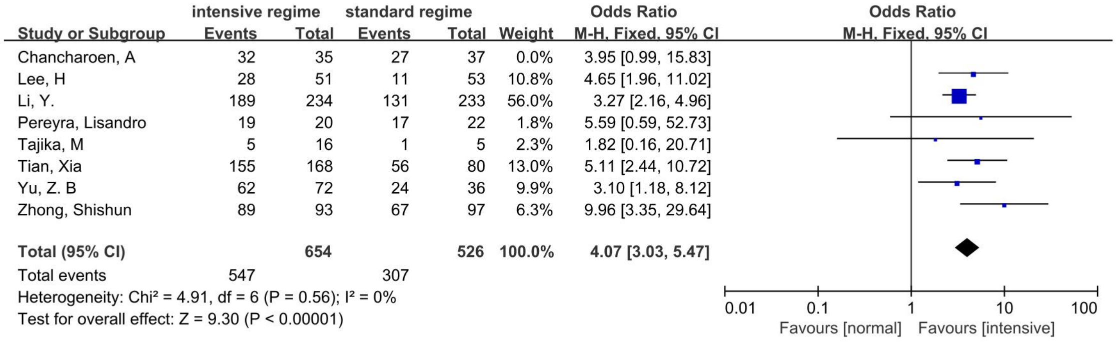

Figure 1. After removing the study of Chancharoen, A, the estimated effect for primary outcome was significantly higher in intensive regimen (OR 4.07, 95 % CI 3.03 to 5.47) .

Study or Subgroup	intensive regime Events	intensive regime Total	standard regime Events	standard regime Total	Weight	Odds Ratio M-H, Fixed, 95% CI
Chancharoen, A	32	35	27	37	5.3%	3.95 [0.99, 15.83]
Lee, H	28	51	11	53	0.0%	4.65 [1.96, 11.02]
Li, Y.	189	234	131	233	59.4%	3.27 [2.16, 4.96]
Pereyra, Lisandro	19	20	17	22	1.9%	5.59 [0.59, 52.73]
Tajika, M	5	16	1	5	2.5%	1.82 [0.16, 20.71]
Tian, Xia	155	168	56	80	13.8%	5.11 [2.44, 10.72]
Yu, Z. B	62	72	24	36	10.5%	3.10 [1.18, 8.12]
Zhong, Shishun	89	93	67	97	6.6%	9.96 [3.35, 29.64]
**Total (95% CI)**		**638**		**510**	**100.0%**	**4.00 [2.94, 5.43]**
Total events	551		323			

Heterogeneity: Chi² = 4.77, df = 6 (P = 0.57); I² = 0%
Test for overall effect: Z = 8.84 (P < 0.00001)

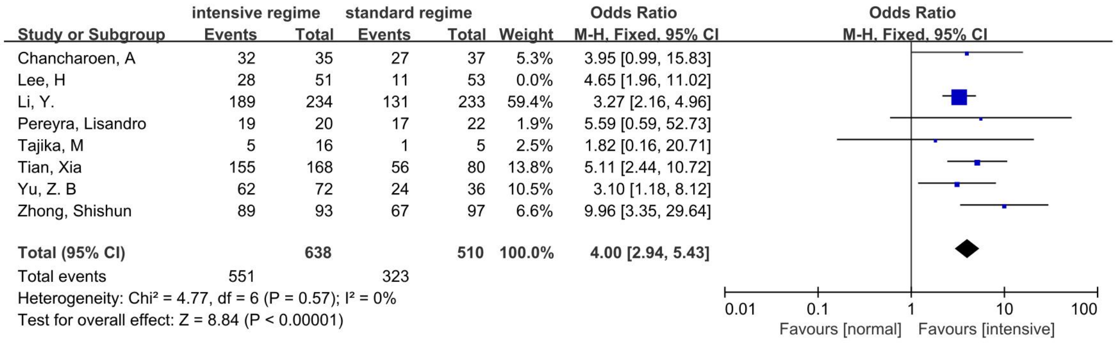

Figure 2. After removing the study of Lee, H, the estimated effect for primary outcome was significantly higher in intensive regimen (OR 4.00, 95 % CI2.94 to 5.43) .

Study or Subgroup	intensive regime Events	intensive regime Total	standard regime Events	standard regime Total	Weight	Odds Ratio M-H, Fixed, 95% CI
Chancharoen, A	32	35	27	37	10.2%	3.95 [0.99, 15.83]
Lee, H	28	51	11	53	22.0%	4.65 [1.96, 11.02]
Li, Y.	189	234	131	233	0.0%	3.27 [2.16, 4.96]
Pereyra, Lisandro	19	20	17	22	3.7%	5.59 [0.59, 52.73]
Tajika, M	5	16	1	5	4.7%	1.82 [0.16, 20.71]
Tian, Xia	155	168	56	80	26.6%	5.11 [2.44, 10.72]
Yu, Z. B	62	72	24	36	20.1%	3.10 [1.18, 8.12]
Zhong, Shishun	89	93	67	97	12.8%	9.96 [3.35, 29.64]
**Total (95% CI)**		**455**		**330**	**100.0%**	**4.97 [3.31, 7.45]**
Total events	390		203			

Heterogeneity: Chi² = 3.29, df = 6 (P = 0.77); I² = 0%
Test for overall effect: Z = 7.74 (P < 0.00001)

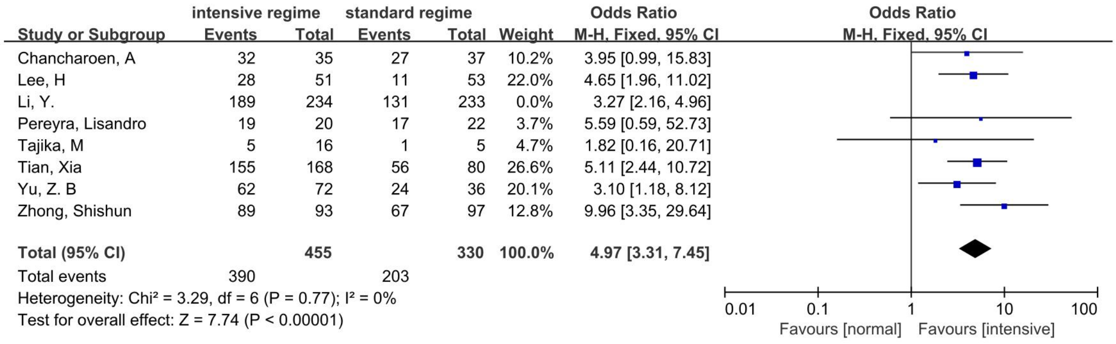

Figure 3. After removing the study of Li, Y, the estimated effect for primary outcome was significantly higher in intensive regimen (OR 4.97, 95 % CI 3.31 to 7.45) .

Study or Subgroup	intensive regime Events	intensive regime Total	standard regime Events	standard regime Total	Weight	Odds Ratio M-H, Fixed, 95% CI
Chancharoen, A	32	35	27	37	4.8%	3.95 [0.99, 15.83]
Lee, H	28	51	11	53	10.5%	4.65 [1.96, 11.02]
Li, Y.	189	234	131	233	54.2%	3.27 [2.16, 4.96]
Pereyra, Lisandro	19	20	17	22	0.0%	5.59 [0.59, 52.73]
Tajika, M	5	16	1	5	2.3%	1.82 [0.16, 20.71]
Tian, Xia	155	168	56	80	12.6%	5.11 [2.44, 10.72]
Yu, Z. B	62	72	24	36	9.5%	3.10 [1.18, 8.12]
Zhong, Shishun	89	93	67	97	6.1%	9.96 [3.35, 29.64]
**Total (95% CI)**		**669**		**541**	**100.0%**	**4.04 [3.01, 5.40]**
Total events	560		317			

Heterogeneity: Chi² = 4.82, df = 6 (P = 0.57); I² = 0%
Test for overall effect: Z = 9.37 (P < 0.00001)

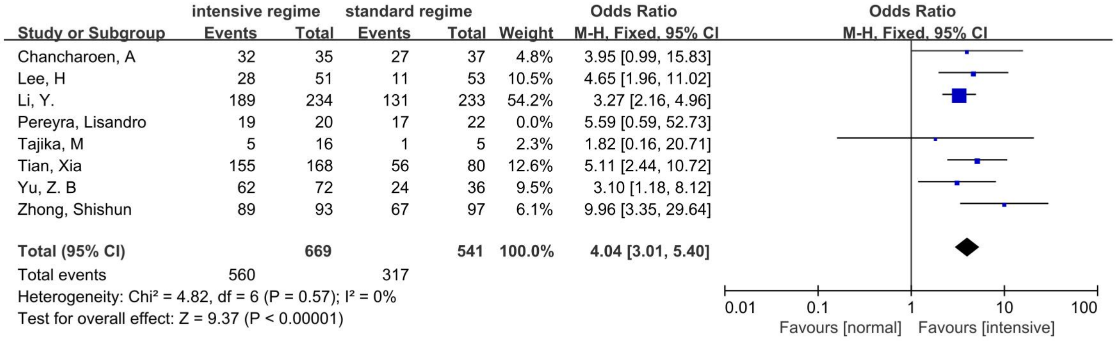

Figure 4. After removing the study of Pereyra, Lisandro, the estimated effect for primary outcome was significantly higher in intensive regimen (OR 4.04, 95 % CI 3.01 to 5.40) .

Study or Subgroup	intensive regime Events	intensive regime Total	standard regime Events	standard regime Total	Weight	Odds Ratio M-H, Fixed, 95% CI
Chancharoen, A	32	35	27	37	4.9%	3.95 [0.99, 15.83]
Lee, H	28	51	11	53	10.5%	4.65 [1.96, 11.02]
Li, Y.	189	234	131	233	54.5%	3.27 [2.16, 4.96]
Pereyra, Lisandro	19	20	17	22	1.7%	5.59 [0.59, 52.73]
Tajika, M	5	16	1	5	0.0%	1.82 [0.16, 20.71]
Tian, Xia	155	168	56	80	12.7%	5.11 [2.44, 10.72]
Yu, Z. B	62	72	24	36	9.6%	3.10 [1.18, 8.12]
Zhong, Shishun	89	93	67	97	6.1%	9.96 [3.35, 29.64]
**Total (95% CI)**		**673**		**558**	**100.0%**	**4.11 [3.07, 5.50]**
Total events	574		333			

Heterogeneity: Chi² = 4.51, df = 6 (P = 0.61); I² = 0%
Test for overall effect: Z = 9.51 (P < 0.00001)

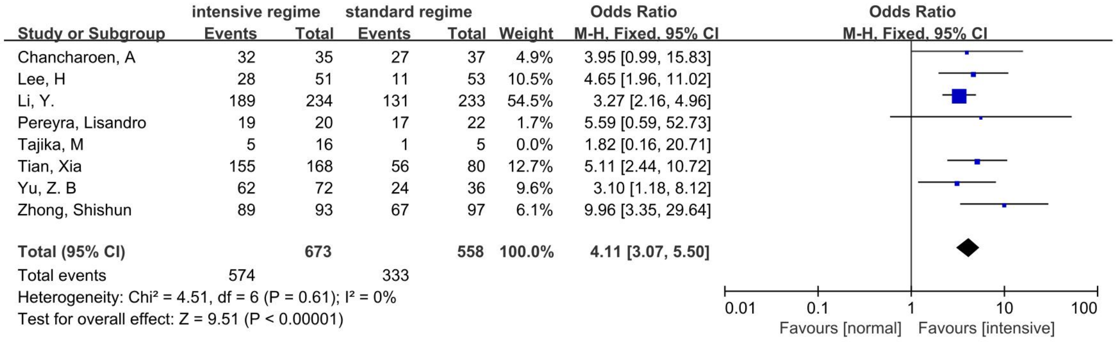

Figure 5. After removing the study of Tajika, M, the estimated effect for primary outcome was significantly higher in intensive regimen (OR 4.11, 95 % CI 3.07 to 5.50) .

Study or Subgroup	intensive regime Events	intensive regime Total	standard regime Events	standard regime Total	Weight	Odds Ratio M-H, Fixed, 95% CI
Chancharoen, A	32	35	27	37	5.4%	3.95 [0.99, 15.83]
Lee, H	28	51	11	53	11.7%	4.65 [1.96, 11.02]
Li, Y.	189	234	131	233	60.9%	3.27 [2.16, 4.96]
Pereyra, Lisandro	19	20	17	22	2.0%	5.59 [0.59, 52.73]
Tajika, M	5	16	1	5	2.5%	1.82 [0.16, 20.71]
Tian, Xia	155	168	56	80	0.0%	5.11 [2.44, 10.72]
Yu, Z. B	62	72	24	36	10.7%	3.10 [1.18, 8.12]
Zhong, Shishun	89	93	67	97	6.8%	9.96 [3.35, 29.64]
**Total (95% CI)**		**521**		**483**	**100.0%**	**3.91 [2.86, 5.36]**
Total events	424		278			

Heterogeneity: Chi² = 4.40, df = 6 (P = 0.62); I² = 0%
Test for overall effect: Z = 8.52 (P < 0.00001)

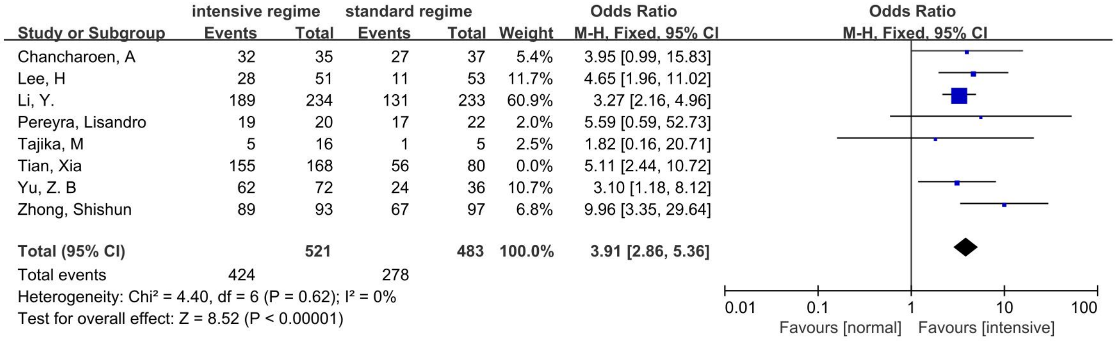

Figure 6. After removing the study of Tian, Xia, the estimated effect for primary outcome was significantly higher in intensive regimen (OR 3.91, 95 % CI 2.86 to 5.36) .

Study or Subgroup	intensive regime Events	intensive regime Total	standard regime Events	standard regime Total	Weight	Odds Ratio M-H, Fixed, 95% CI
Chancharoen, A	32	35	27	37	5.2%	3.95 [0.99, 15.83]
Lee, H	28	51	11	53	11.3%	4.65 [1.96, 11.02]
Li, Y.	189	234	131	233	58.8%	3.27 [2.16, 4.96]
Pereyra, Lisandro	19	20	17	22	1.9%	5.59 [0.59, 52.73]
Tajika, M	5	16	1	5	2.4%	1.82 [0.16, 20.71]
Tian, Xia	155	168	56	80	13.7%	5.11 [2.44, 10.72]
Yu, Z. B	62	72	24	36	0.0%	3.10 [1.18, 8.12]
Zhong, Shishun	89	93	67	97	6.6%	9.96 [3.35, 29.64]
**Total (95% CI)**		**617**		**527**	**100.0%**	**4.16 [3.07, 5.64]**
Total events	517		310			

Heterogeneity: Chi² = 4.63, df = 6 (P = 0.59); I² = 0%

Test for overall effect: Z = 9.20 (P < 0.00001)

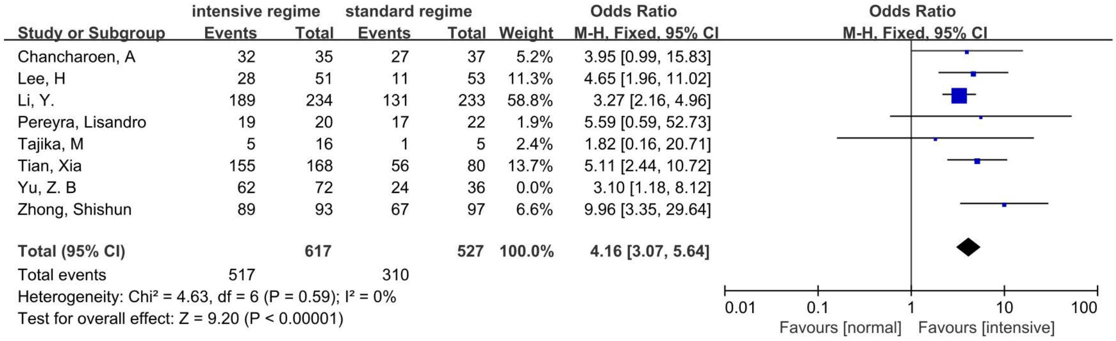

Figure 7. After removing the study of Yu, Z.B, the estimated effect for primary outcome was significantly higher in intensive regimen (OR 4.16, 95 % CI 3.07 to 5.64) .

Study or Subgroup	intensive regime Events	intensive regime Total	standard regime Events	standard regime Total	Weight	Odds Ratio M-H, Fixed, 95% CI
Chancharoen, A	32	35	27	37	5.1%	3.95 [0.99, 15.83]
Lee, H	28	51	11	53	10.9%	4.65 [1.96, 11.02]
Li, Y.	189	234	131	233	56.7%	3.27 [2.16, 4.96]
Pereyra, Lisandro	19	20	17	22	1.8%	5.59 [0.59, 52.73]
Tajika, M	5	16	1	5	2.4%	1.82 [0.16, 20.71]
Tian, Xia	155	168	56	80	13.2%	5.11 [2.44, 10.72]
Yu, Z. B	62	72	24	36	10.0%	3.10 [1.18, 8.12]
Zhong, Shishun	89	93	67	97	0.0%	9.96 [3.35, 29.64]
**Total (95% CI)**		**596**		**466**	**100.0%**	**3.69 [2.73, 4.99]**
Total events	490		267			

Heterogeneity: Chi² = 1.93, df = 6 (P = 0.93); I² = 0%

Test for overall effect: Z = 8.45 (P < 0.00001)

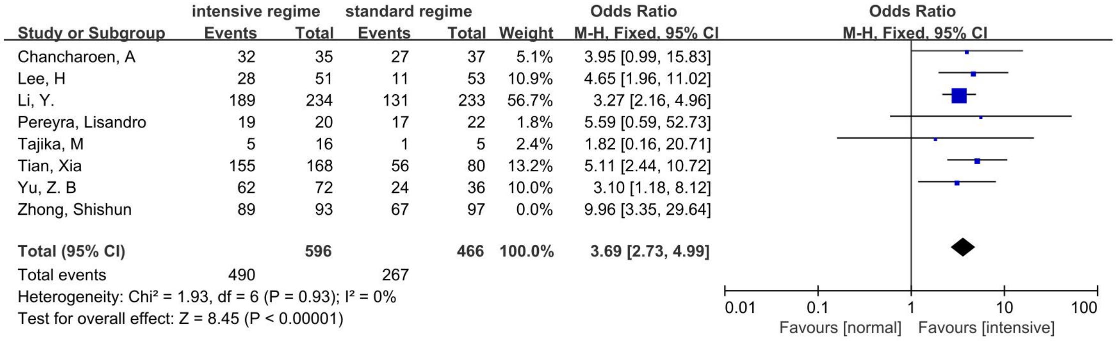

Figure 8. After removing the study of Zhong, Shishun, the estimated effect for primary outcome was significantly higher in intensive regimen (OR 3.69, 95 % CI 2.73 to 4.99) .
